# Pokeweed Antiviral Protein: Its Cytotoxicity Mechanism and Applications in Plant Disease Resistance

**DOI:** 10.3390/toxins7030755

**Published:** 2015-03-06

**Authors:** Rong Di, Nilgun E. Tumer

**Affiliations:** Department of Plant Biology and Pathology, Rutgers, the State University of New Jersey, 59 Dudley Road, New Brunswick, NJ 08901, USA; E-Mail: tumer@aesop.rutgers.edu

**Keywords:** pokeweed antiviral protein, ribosome inactivating protein, rRNA depurination, apoptosis, ribosomal protein, broad spectrum disease resistance

## Abstract

Pokeweed antiviral protein (PAP) is a 29 kDa type I ribosome inactivating protein (RIP) found in pokeweed plants. Pokeweed produces different forms of PAP. This review focuses on the spring form of PAP isolated from *Phytolacca americana* leaves. PAP exerts its cytotoxicity by removing a specific adenine from the α-sarcin/ricin loop of the large ribosomal RNA. Besides depurination of the rRNA, PAP has additional activities that contribute to its cytotoxicity. The mechanism of PAP cytotoxicity is summarized based on evidence from the analysis of transgenic plants and the yeast model system. PAP was initially found to be anti-viral when it was co-inoculated with plant viruses onto plants. Transgenic plants expressing PAP and non-toxic PAP mutants have displayed broad-spectrum resistance to both viral and fungal infection. The mechanism of PAP-induced disease resistance in transgenic plants is summarized.

## 1. Introduction

Pokeweed antiviral protein (PAP), purified from the leaves of pokeweed plant (*Phytolacca americana*), was found to be a potent inhibitor of eukaryotic protein synthesis [1] and several plant and animal viruses [2,3] more than four decades ago [4]. PAP’s inhibitor capacity has been attributed to its enzymatic activity on the ribosomes. PAP removes a specific adenine residue from the highly conserved α-sarcin/ricin loop (SRL) in the 28S rRNA of the eukaryotic ribosome [5] by a process termed depurination, which is characteristic of a group of proteins called ribosome inactivating proteins (RIPs). PAP belongs to type I RIPs, which consist of a single basic polypeptide chain of approximately 30 kDa. Ricin from the castor bean (*Ricinus communis*) is the type member of the type II RIPs, which consist of a single active A-chain covalently linked to a galactose-binding B-chain [6]. Type II RIPs also include Shiga toxins produced by bacteria *Shigella* *dysenteriae* and enterohemorrhagic *Escherichia coli* (EHEC) [7]. Both type I and type II RIPs are secretory proteins. Other types of RIPs have also been described that are not secretory proteins, including the atypical maize RIP MOD with an internal inactivation loop [8,9,10]. The barley JIP60 (jasmonate-induced protein 60), an inducible protein, has been classified as a type III RIP with two domains: an NH_2_-terminal domain related to RIPs and an extended COOH-terminal domain similar to the eukaryotic translation initiation factor 4E (eIF4E) [11,12,13].

We have studied PAP since the 1990s, focusing on the mechanism of its cytotoxicity and its applications to agriculture. The general characteristics, enzymatic function, antiviral activity of PAP and its applications in medicine have been reviewed in 1999 [14] and in 2004 [15]. Since then, great advances have been made in understanding the cytotoxicity of PAP and PAP-induced disease resistance in plants. These two aspects of PAP will be the focus of this review.

## 2. Different Forms of PAP

**Figure 1:**
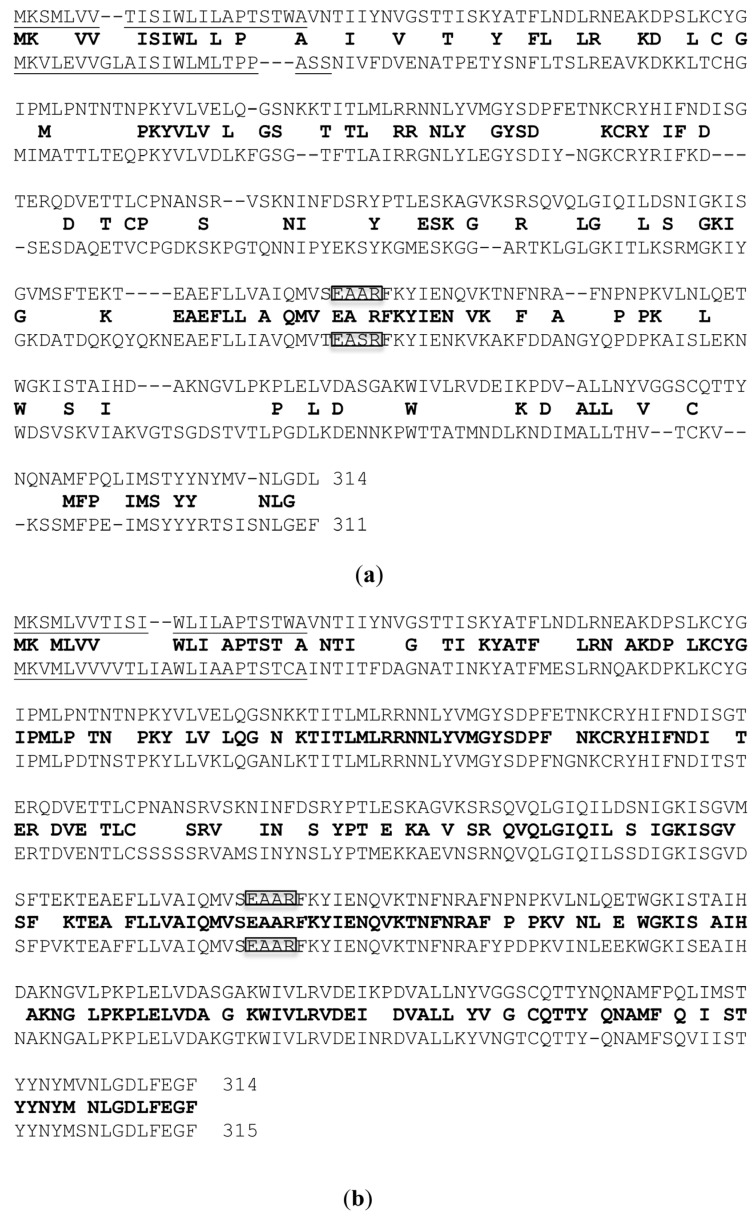
Amino acid alignments between pokeweed antiviral protein (PAP) and PAP II (**a**) PAP and PAP-S (**b**) using BLAST. The consensus amino acids are listed in the middle in bold between PAP on the top and PAP II or PAP-S at the bottom. The *N*-terminal signal peptides are underlined. The amino acids in the active sites are boxed.

Several different isoforms of PAP have been isolated from *Phytolacca americana*, namely PAP (sometimes referred as PAP I) from the spring leaves, PAP II and PAP III from the early summer and late summer leaves [16]. Additionally, PAP-S has been purified from pokeweed seeds in 1982 [17]. Later on, two isoforms of PAP-S (PAP-S1 and PAP-S2) from pokeweed seeds were described [18]. Genes encoding PAP [19], PAP II [20] and PAP-S have been cloned and characterized. The complete cDNA sequences for PAP, PAP II and PAP-S are available from GenBank with the following accession numbers: AR009535, X86085 and X98079. PAP, PAP II and PAP-S genes are 942, 933 and 945 nucleotides long and encode 314, 311 and 315 amino acid proteins respectively. The molecular weight of PAP proteins is approximately 29 kDa. The amino acid sequences of PAP, PAP II and PAP-S have been compared in 1997 [21]. The amino acid sequence differences between PAP and the other forms, PAPII and PAP-S are illustrated in Figure 1.

PAP shares 39% and 76% homology with PAP II and PAP-S respectively. It is interesting to note that although both PAP and PAP II are found in pokeweed leaves, PAP is more closely related to PAP-S than to PAP II. It has been shown that both PAP and PAP-S genes do not contain introns while PAP II contains two exons separated by one intron [20,21]. The *N*-terminal 22, 23 and 24 amino acids have been identified as the putative signal peptides for PAP, PAP II and PAP-S respectively [20,21,22]. PAP, PAP II and PAP-S are considered as secretory RIPs. It was shown earlier by electron microscopy that PAP-specific antibody detected PAP bound within the cell wall matrix of leaf mesophyll cells of *P. americana*, indicating that PAP is secreted [23]. A recent study showed that PAP formed homodimer complexes in the cytosol of pokeweed cells, and its monomeric form was found in the apoplast [24]. The PAP homodimer was demonstrated to be much less active on rRNA compared to the monomeric PAP. Thus, it was concluded that PAP uses the homodimerization mechanism to avoid depurinating the pokeweed rRNA [24].

**Figure 2:**
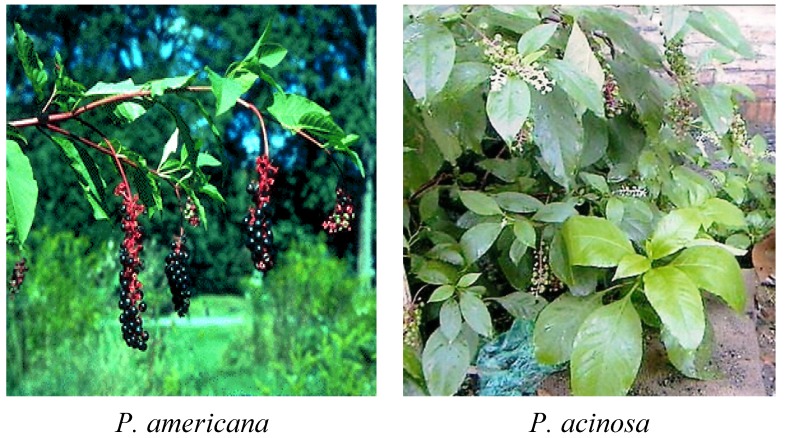
Pictures of perennial decidious *Phytolacca americana* from New Jersey, central eastern USA and perennial evergreen *Phytolacca acinosa* from Fujian Province, southeast China.

PAP has also been isolated and characterized from pokeweed plant found in southwest China, *Phytolacca acinosa* Roxh*.* Two accessions are present in the GenBank, PAPa1 (#AY603353) and PAPa2 (#AY603354) with partial sequences starting from the first amino acid to the 238th of the mature protein. Amino acid alignments showed that PAPa1 and PAPa2 are 98.8% and 95% homologous to PAP. Based on the sequence of PAP (accession #AR009535), PCR primers were designed and used to clone the PAP gene from genomic DNA isolated from the leaves of *Phytolacca acinosa* found in the subtropical Fujian Province in southeast China (Figure 2). The full-length PAP gene was cloned from *Phytolacca acinosa* and named ChPAP (Chinese Pokeweed Antiviral Protein). Amino acid alignment indicates that ChPAP differs from PAP by only one amino acid. 

## 3. Cytotoxicity of PAP

### 3.1. PAP Cytotoxicity in Plants

**Figure 3:**
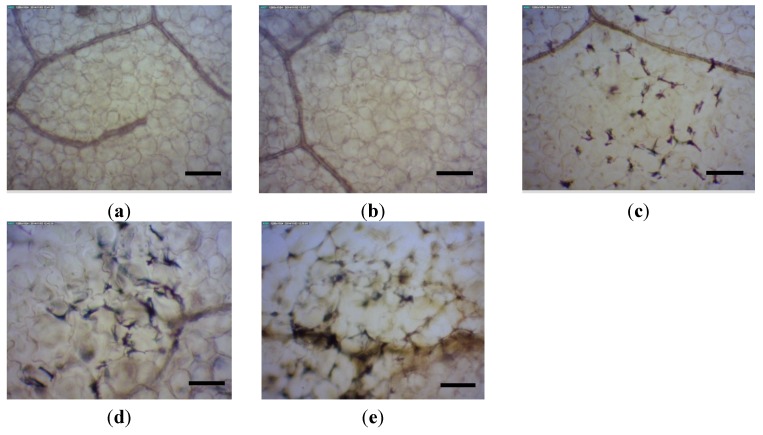
Trypan blue staining of tobacco leaves. (**a**) 8 week old wt *N. tabacum*, 10×; (**b**) 8 week old symptomless transgenic tobacco, 10×; (**c**) 8 week old symptomatic transgenic tobacco, 10×; (**d**) 8 week old symptomatic transgenic tobacco, 20× and (**e**) *N. tabacum* NN 7 days post TMV inoculation, 20×. Bars in (**a**) to (**c**) = 100 µm. Bars in (**d**) to (**e**) = 50 µm.

PAP removes a specific adenine from the SRL of the large rRNA and inhibits translation, leading to cell death. The cytotoxicity of wild type (wt) PAP made it very difficult to regenerate transgenic tobacco pants (*Nicotiana tabacum* cv. Samsun) [22]. As reported [22], the transformation efficiency was only 0.7%, compared to 7%–18% for the vector control without the PAP gene. When a variant of PAP (PAPv) containing mutations of L20R and Y49H was used to transform tobacco, 3.7% transformation efficiency was obtained, indicating that PAPv was less toxic than wt PAP. It was shown that high PAP-expressing transgenic tobacco plants showed mottled symptoms on their leaves. They were stunted and their seeds were not viable. However, the low PAP-expressing plants were indistinguishable from non-transformed plants and they produced viable seeds [22]. When the leaves of PAPv expressing transgenic tobacco plants were stained with lactophenol-trypan blue solution (2.5 mg/mL trypan blue, 25% lactic acid, 25% phenol and 25% glycerol) [25] and visualized with a compound microscope, the symptomatic leaves showed the trypan blue staining pattern characteristic of programed cell death. The pattern observed was similar to the necrotic local lesions produced when *N. tabacum* cv. Samsun plants containing the N gene [26] were infected with the tobacco mosaic virus (TMV) (Figure 3). This pattern of trypan blue staining has also been documented in plants showing the hypersensitive response to bacterial infection [25]. These results showed that PAP expressed in transgenic tobacco plants is toxic and causes cell death.

### 3.2. PAP Cytotoxicity in Yeast

A yeast system has been developed as a model to study the cytotoxicity of PAP. The full-length cDNA of PAP (accession #AR009535) was cloned under the control of the galactose inducible *GAL1* promoter and transformed into *Saccharomyces cerevisiae* strain W303 (*Mata ade2-1 trp1-1 ura3-1 leu2-3 112 his3-11 15 can1-100*) [27]. PAP was properly processed in yeast cells and the induction of PAP expression was lethal to yeast. To understand how PAP interacts with rRNA and causes cell death, the PAP expression plasmid was mutagenized with hydroxylamine and re-transformed into yeast cells. Yeast cells harboring non-toxic PAP mutants were identified as those that could grow after induction of expression on galactose. One of the non-toxic mutants identified was PAP_E176V_, which abolished PAP cytotoxicity. The Glu-176 in PAP is equivalent to Glu-177 of ricin A chain (RTA) which had been previously implicated as one of the key residues at the active site [28]. PAP and RTA share the same EAAR residues in their active sites (Figure 1) [29]. Glu-177 (E) and Arg-180 (R) residues of RTA were identified as critical for its enzymatic activity [30].

Extensive site-directed mutagenesis of PAP was carried out. Over 30 mutations at the *N*-terminus, central domain and the *C*-terminus of PAP were generated, transformed into yeast and characterized [31]. The dual primer extension method was used to measure the depurination of rRNA, and the *in vivo* (^35^S)-methionine incorporation assay was used to assess the inhibition of translation in yeast cells transformed with the PAP mutants. Besides PAP_E176V_, PAP_G75D_ mutation in the central domain led to loss of depurination activity and cytotoxicity. Additionally, both PAP_Y16M_ and PAP_T18M_ mutations at the *N*-terminus were not toxic to yeast cells and these mutants did not depurinate the rRNA. Interestingly, the following PAP mutations in the central domain, PAP_N70A_, PAP_L71R_, PAP_Y72A_, PAP_V73E_, PAP_Y76A_ and PAP_Y123A_ and PAP_Y123I_, led to loss of cytotoxicity, but not rRNA depurination. These results indicated that cytotoxicity of PAP was lost before ribosome depurination ability, suggesting that ribosome depurination was not the only factor contributing to cytotoxicity [31]. 

Recent results showed that yeast cells expressing PAP exhibited apoptotic-like features, such as nuclear fragmentation and production of reactive oxygen species (ROS). *Arabidopsis thaliana* Bax inhibitor-1, a plant antiapoptotic protein, which inhibits Bax induced cell death reduced the cytotoxicity of PAP in yeast, without affecting ribosome depurination and translation inhibition [32]. These results provided further evidence that ribosome depurination and translation inhibition by PAP could be separated from cytotoxicity.

### 3.3. Mechanism of PAP Cytotoxicity

#### 3.3.1. *N*-glycosidase Activity

Endo and Tsurugi reported in 1987 that RTA removed a single adenine from position 4324 in the 28S rRNA of rat liver ribosomes [33]. It was demonstrated that RTA cleaved the *N*-glycosidic bond of A^4324^ of the 28S rRNA in a hydrolytic fashion, thus defining RIPs as ribosome-specific *N*-glycosidases (EC 3.2.2.22) [33]. Based on structural analysis, Monzingo and Robertus [34] proposed the following enzymatic action for ricin: Tyr^80^ (equivalent to PAP Tyr^72^) and Tyr^123^ (equivalent to PAP Tyr^123^) sandwich the target adenine ring, the negative charge of Glu^177^ (equivalent to PAP Glu^176^) stabilizes the positive oxycarbonium ion on ribose in the transition state, and Arg^180^ (equivalent to PAP Arg^179^) stabilizes the anion on the leaving adenine by protonating it at the N-3 position. Later on, Rajamohan *et al.* used site-directed mutagenesis, binding assays and surface plasmon resonance (SPR) to provide experimental evidence that Tyr^72^, Tyr^123^, Glu^176^ and Arg^179^ are catalytic residues that participate in the binding of PAP to the target tetraloop structure of the SRL [35].

Since PAP is a type I RIP, it lacks a sugar-binding moiety, such as the ricin B-chain. Recently, using fluorescence spectroscopy, Nakashima *et al.* demonstrated that the Trp^208^ at the *C*-terminus of PAP cooperated with Tyr^72^ and bound strongly to N-acetylglucosamine (NAG, a monosaccharide derivative of glucose) [36]. Since Trp^208^ is considered critical for the enzymatic activity of RIPs, the authors concluded that the sugar-binding may induce a conformational change near the active site of PAP.

RIPs are active on eukaryotic rRNA. PAP, however, was shown to depurinate both the A^4324^ of the eukaryotic ribosome and the A^2660^ of the *Escherichia coli (E. coli)* ribosome [37]. After the crystal structure of PAP was refined at room temperature [38] and lower temperatures [39], Kurinov *et al.* demonstrated that PAP could release not only adenine but also guanine from *Escherichia coli* ribosomes at a 20 times slower rate [40]. Although these studies provided information about the *N*-glycosidase activity of PAP, they did not address its ribosome specificity or its ability to depurinate the eukaryotic and prokaryotic ribosomes.

#### 3.3.2. Interaction with Ribosomal Proteins

To answer the questions above and to elucidate the mechanism of PAP’s interaction with the ribosome, the interaction of PAP with ribosomal proteins has been examined [41]. Ribosomes of yeast cells harboring the *mak8-1* allele of the large ribosomal subunit protein L3 (RPL3) were shown to be resistant to PAP depurination [42]. Since the highly conserved RPL3 participates in the formation of the peptidyltransferase center [43,44] and is essential for the catalysis of peptide bond formation [45], RPL3 was shown to be critical for the binding of PAP to ribosomes and the subsequent rRNA depurination. It was shown later by SPR affinity analysis that PAP active site cleft residues Asn^69^, Asn^70^ and Asp^92^ are involved in binding to RPL3 [46].

The interaction between PAP and RPL3 was further examined in transgenic tobacco plants. As mentioned in 3.1, wt PAP and PAP_V_ expressed in transgenic tobacco plants are toxic to tobacco cells [22]. However, when tobacco (*N. tabacum* cv. Samsun) was transformed with a construct containing both the full-length yeast RPL3 and wt PAP, fertile transgenic plants were produced [47]. The first two leaves of the transgenic tobacco plants showed only mild mottle symptoms and their ribosomes were partially depurinated. However, transgenic tobacco plants expressing the *N*-terminal 99 amino acids of yeast L3 and wt PAP were phenotypically normal compared to non-transformed tobacco plants and were fertile. Their ribosomes were not depurinated even though PAP was associated with the ribosomes. This work suggested that PAP may interact with the *N*-terminus of RPL3 in transgenic tobacco plants.

To further study the interaction between PAP and RPL3, the yeast model was used. Yeast cells transformed with both wt PAP and the *N*-terminal 99 amino acids of RPL3 (L3Δ99) showed markedly reduced growth inhibition and rRNA depurination compared to cells transformed with PAP alone [48]. Additionally, it was shown that the *N*-terminal 21 amino acids of RPL3 (L3Δ21) were sufficient to reduce the cytotoxicity of PAP in yeast cells [48]. By SPR analysis, PAP interacted with a 27-mer RNA fragment corresponding to the SRL with a binding affinity of 0.365 nM (unpublished data), which was similar to the 0.2 nM affinity published earlier [35]. We also showed that the affinity of PAP for a 40-mer RNA fragment corresponding to L3Δ was 4.91 nM. Furthermore, it was shown that PAP interacted with the yeast L3Δ21 peptide with an affinity of 24.8 nM [48]. These results indicate that PAP interacts with both and RNA fragment and a peptide corresponding to the *N*-terminus of RPL3. Since RPL3 is highly conserved among different species, the interaction between PAP and RPL3 may partially account for the non-selective cytotoxicity of PAP to rRNA from different organisms. It has been shown that ricin [49], trichosanthin [50,51], Shiga toxin 1 [49,52] and maize RIP MOD [53] interact with the P proteins of the ribosomal stalk. However, this is not the case for PAP [49,51]. Furthermore, it was shown that residues Gly^209^–Lys^225^ at the PAP *C*-terminal domain were critical for its N-glycosidase activity on prokaryotic ribosomes [54]. Since the stalk structure differs between eukaryotic and prokaryotic ribosomes, it has been postulated that the ability of these RIPs to interact with the P protein motif originated during evolution of the eukaryotic elongation factor (EF)-2 [55].

#### 3.3.3. Inhibition of Translation

As early as in 1976 it was shown that protein synthesis was inhibited upon rRNA depurination by RIPs and the effect occurred at the peptide elongation step [56]. Roles of the elongation factors and ribosomes during protein synthesis have been reviewed extensively [57,58,59]. During the elongation step in protein synthesis, EF1 brings aminoacyl-tRNA to the A-site of the ribosome and after codon and anti-codon recognition, EF1 is released. The rRNA catalyzes the peptide formation and transfers the peptidyl-tRNA from the P-site of the ribosome to the A-site. It has been shown that the translocation of peptidyl tRNA from the P-site to A-site by EF2 was inhibited after rRNA was depurinated by RIPs [60]. It was recently demonstrated that PAP depurinated the rRNA prior to the binding of aminoacyl-tRNA to the ribosomal A-site [61].

#### 3.3.4. Other Enzymatic Activity

Different substrates including DNA, RNA and poly (A) RNA have been described for the alternative enzymatic activity of RIPs [62,63]. PAP_G75D_ and PAP_W237*_ (* denotes stop codon) isolated from transgenic tobacco plants did not depurinate the rabbit reticulocyte rRNA; however, they depurinated the capped viral RNAs and inhibited the translation of brome mosaic virus (BMV) and potato virus X (PVX) RNAs [64]. It was shown that PAP’s inhibition of capped BMV RNA translation was overcome in the presence of the cap analog m^7^GpppG, indicating that PAP recognized the cap structure [64]. Subsequently, PAP was shown to bind to the cap structure of eukaryotic mRNA and depurinate the mRNA downstream of the cap [65]. It was demonstrated that PAP interacted with the eukaryotic initiation factor (eIF)4G and eIFiso4G in the wheat germ lysate, providing a mechanism for PAP to access both uncapped and capped RNAs [66]. Further evidence for PAP binding to the cap analog m^7^GTP was provided by fluorescence quenching experiments [67].

PAP regulated its own mRNA stability by a mechanism that involved depurination [48,68]. These data suggested that rRNA is not the only substrate for PAP, capped mRNAs and uncapped RNAs are subject to inactivation by PAP. Additionally, double-stranded (ds) supercoiled DNA could be cleaved by PAP [69]. The active site required for rRNA depurination was also required for DNA cleavage, as the non-rRNA depurinating PAP_E176V_ could not cleave the dsDNA [69]. The dsDNA treated with PAP contained apurinic/apyrimidinic (AP) sites due to the removal of adenine [69]. The same phenomenon was observed with different RIPs including gelonin, momordin I, PAP-S and saporin-S6 [70].

#### 3.3.5. *C*-Terminal Involvement in PAP Processing

Type II RIPs such as ricin and Shiga toxins use their lectin chain to bind to receptors on cells [71,72,73,74]. They are internalized by endocytosis and undergo retrograde transport via the Golgi complex to reach the endoplasmic reticulum (ER) lumen. After processing, they are thought to retro-translocate into the cytosol using the ER-associated degradation (ERAD) pathway. PAP does not contain a lectin chain. It is synthesized as a 314-amino acid precursor containing a 22 residue *N*-terminal signal sequence and a 29 residue *C*-terminal extension [75]. The *N*-terminal 22 amino acids of PAP direct PAP to the apoplast in pokeweed plants [22,23]. When total protein from yeast cells transformed with wt PAP was electrophoresed on the SDS-polyacrylamide gel, two protein forms were observed [76]. It has been shown that both PAP forms associate with the ER in yeast cells, and the smaller PAP form may retrotranslocate into the cytosol [76].

When the *C*-terminus (_249_VALLNYVGGSCQTT_262_) of mature PAP was sequentially truncated, cytotoxicity was lost before rRNA depurination in yeast [31]. Specifically, PAP_N253*_ mutant was toxic, while PAP_L252*_ was not toxic even though it depurinated ribosomes. PAP’s *C*-terminal motif was similar to those found in ricin and Shiga toxins [75]. Further analysis showed that the *C*-terminal amino acids were critical for the processing of PAP and the accumulation of mature PAP in the cytosol. Thus, they affected depurination of the rRNA in the cytosol and the cytotoxicity of PAP [75].

## 4. Applications of PAP in Plant Disease Resistance

### 4.1. Against Plant Viruses

PAP was first discovered in 1925 for its activity against plant viruses [77]. It was later found that when pokeweed extracts were co-inoculated with a plant virus onto the leaves of susceptible plants, PAP in the crude extract could protect plants from different viruses, such as southern bean mosaic virus [2], cucumber mosaic virus (CMV) [3] and TMV [78]. In 1990s when plant biotechnology became possible, the great potential of PAP as a plant pathogen inhibitor was realized.

Lodge *et al.* [22] showed for the first time that tobacco and potato plants transformed with wt PAP and PAP_L20R/Y49H_ under the control of CaMV 35S promoter were resistant to potato virus X (PVX), potato virus Y (PVY) and CMV [22]. These results indicated broad spectrum virus resistance, in contrast to viral coat protein-mediated resistance, which was specific to the virus the coat protein gene was derived from. PAP was enriched in the intercellular fluid of transgenic plants and conferred viral resistance by inhibiting an early stage of infection. It was hypothesized that upon infection PAP might enter the host cells along with the virus and depurinate the host rRNA. Since viruses depend on the host machinery for their replication, ribosome-damaged host cells will not be able to support virus replication [22]. This hypothesis was supported by the work of Chen *et al.* [79], which showed that when PAP and TMV were co-inoculated onto tobacco plants, ribosomes were depurinated as early as 5 min after inoculation and the inhibition of virus infection and ribosome depurination were positively correlated with PAP concentration [79]. Later, Tumer *et al.* reported that ribosomes of PAP_L20R/Y49H_ expressing transgenic tobacco plants were depurinated [80].

However, when Tumer *et al.* [80] transformed tobacco with the nontoxic *C*-terminal deletion mutant PAP_W237*_ and the active site mutant PAP_E176V_, they found that ribosomes from these transgenic tobacco plants were not depurinated. The extracts from PAP_W237*_ expressing transgenic plants protected tobacco plants from PVX infection, while the extracts from PAP_E176V_ expressing plants did not. Furthermore, they found that transgenic tobacco plants expressing PAP_W237*_ were resistant to PVX infection, while plants expressing PAP_E176V_ were not resistant. These data indicated that the *C*-terminus of PAP was required for toxicity and depurination of ribosomes, but not for antiviral activity, suggesting that host ribosome depurination was not the only mechanism for PAP-induced virus resistance [80].

It is mentioned earlier that non-depurinating mutants PAP_G75D_ and PAP_W237*_ isolated from transgenic tobacco plants could depurinate the capped BMV and PVX viral RNAs and inhibit their translation *in vitro* [64]. PAP could inhibit translation of uncapped TBSV (tomato bushy stunt virus) and SPMV (satellite panicum mosaic virus) *in vivo* without causing detectable depurination in the viral RNAs [81]. The inhibition of PAP on BMV replication was shown later in barley protoplasts [82]. The inhibition was due to the depurination of BMV RNA by PAP and inhibition of RNA replication and subgenomic RNA transcription [82,83]. These data indicate that besides depurinating host ribosomes, PAP can directly depurinate viral RNA and inhibit virus replication.

### 4.2. Against Plant Fungi

Anti-fungal activities of RIPs have been less frequently described compared to their anti-viral activity. The 30 kDa cytosolic type I RIP from barley was shown to be inactive on plant ribosomes *in vitro* as other type I RIPs from cereal starchy endosperms [84], but capable of modifying fungal ribosomes [85]. Indeed, when this barley endosperm RIP was transformed into tobacco under the control of an inducible promoter, it conferred resistance to *Rhizoctonia solani* without affecting the growth of transgenic tobacco plants [86]. Furthermore, co-expression of the barley RIP with a class-II chitinase resulted in synergistically enhanced resistance to *R. solani* in transgenic tobacco plants [87]. It was proposed that the hydrolytic activity of chitinase could result in an increased uptake of the barley RIP into fungal cells and significantly inhibit the fungal growth [87]. Another cereal endosperm RIP, the maize kernel RIP I, was also shown to be anti-fungal, inhibiting the postdivisional growth of *Aspergillus* spp. in microculture assays [88]. However, the exact mechanism of how endosperm RIPs affect fungal growth is not well understood.

It was found that PAP_L20R/Y49H_- and PAP_W237*_-transgenic plants were resistant to *R. solani* infection [89]. Additionally, expression of both class I (basic) and class II (acidic) isoforms of pathogenesis-related (PR) (PR1 and PR2) proteins was induced in the transgenic tobacco plants [89,90]. However, the salicylic acid (SA) levels were not elevated [89,90] as in the classical systemic acquired resistance (SAR). Subsequently, it was shown that transgenic tobacco plants expressing the non-toxic mutant PAP_G75D_ were resistant to *R. solani* infection [91]. PR2 expression was not elevated in PAP_G75D_-transgenic plants, rather the expression levels of PR1 and wound-inducible protein kinase (WIPK) and protease inhibitor II (PI-II) were up-regulated [91]. The SA level in PAP_G75D_-plants was also not increased [91]. These results suggested that the PAP expressed in transgenic plants induces expression of several PR proteins, likely through an SA-independent pathway [89,90].

### 4.3. Mechanisms of PAP-Induced Disease Resistance

**Figure 4:**
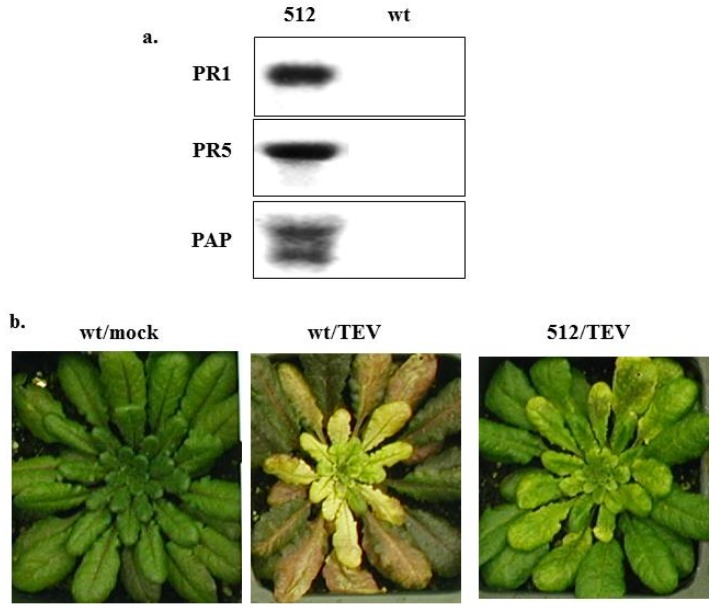
(**a**) Northern blot analysis of PAP_W237*_-transgenic line 512 and wt *Arabidopsis* plants using PR1, PR5 and PAP specific ^32^P-labelling cDNA probes; (**b**) Mock (water) and tobacco etch virus (TEV)-inoculated wt and PAP_W237*_-transgenic line 512 *Arabidopsis* plants.

To further investigate the disease resistance mechanisms induced by PAP, transgenic *Arabidopsis* plants expressing the non-toxic *C*-terminal deletion mutant PAP_W237*_ were produced. Figure 4a shows that PAP was over-expressed in the transgenic line 512 compared to non-transformed wt *Arabidopsis thaliana* Columbia ecotype by Northern blot analysis using PAP cDNA as a probe. Figure 4a also shows that PR1 [92] and PR5 [93] genes were up-regulated in line 512, supporting the early findings in transgenic tobacco plants [89,91]. Line 512 displayed enhanced resistance to a strain of tobacco etch virus (TEV) that causes systemic infection [94] compared to wt *Arabidopsis*, as the transgenic plant only showed very mild yellowing symptoms on its leaves (Figure 4b). The transcript profiling analysis using the Affymetrix^®^ *Arabidopsis* microarrays was conducted. Table 1 lists the genes that were up-regulated in PAP_W237*_ line 512 for more than 3-fold compared to wt *Arabidopsis*. The expression of several auxin-responsive genes was more than 4-fold higher in line 512 than in wt *Arabidopsis*. Notably, the lipoxygenase (lox3, AJ249794) gene that is critical for jasmonic acid (JA)-mediated induced systemic resistance (ISR) [92] was up-regulated by 4.6-fold. The other plant innate immunity and disease resistance-related genes that were up-regulated in line 512 include the AtERF6 ethylene responsive element binding factor (AB013301) [95], PR1 (X96600) [92], PR5K (U48698) [93,96], and thaumatin-like protein (U83490) [97]. We have confirmed the expression of these genes by quantitative RT-PCR (qRT-PCR) analysis using gene-specific primers (data not shown).

**Table 1:** Transcript profiling of PAP_W237*_-transgenic line 512 *vs.* wt *Arabidopsis* using Affymetrix^®^ *Arabidopsis* microarray: Fold change of up-regulated gene expression.

Gene	Gene description	Fold change
U49076	early auxin-induced (IAA20) mRNA	6.1
AF087819	auxin transport protein (PIN6) mRNA	5.7
AF082176	auxin response factor 9 (ARF9) mRNA	5.7
L15448	auxin-responsive protein (IAA1) mRNA	5.7
AF087819	auxin transport protein (PIN6) mRNA	5.3
AL035656	putative auxin-induced protein	4.9
AL035656	small auxin up RNA (SAUR-AC1)	4.9
AL035656	auxin-induced protein-like	4.9
S70188	small auxin up RNA	4.9
AJ249794	lipoxygenase (lox3)	4.6
AB013301	AtERF6 ethylene responsive element binding factor	4.3
AJ012745	RH27 helicase	4.3
Z97341	RNA helicase	3.7
AJ010475	DEAD box RNA helicase, RH28	3.7
Z97337	RNA helicase like protein	3.7
X96600	pathogenesis-related protein 1 (PR1)	3.5
U48698	receptor serine/threonine kinase PR5K (PR5K)	3.5
U83490	thaumatin-like protein	3.2
AL021687	cytochrome P450	3
AL049659	cytochrome P450-like protein	3
AL021636	cytochrome P450-like protein	3
AL049659	cytochrome P450-like protein	3

These data support the previous findings and indicate that PAP elicits a wide range of defense responses in transgenic plants, which may be responsible for the fungal resistance observed in these plants. The transgene approach has been successfully used to confer plant disease resistance. However, there are few examples of transgenes, which can induce broad-spectrum disease resistance. As plants are often infected with multiple pathogens, engineering them with single genes, such as nontoxic PAP variants that can confer broad-spectrum disease resistance may be more advantageous and may have many applications to agriculture.
